# Improved guideline adherence may not overcome evidence gaps in severe trimethoprim-sulfamethoxazole-resistant *Stenotrophomonas maltophilia* infection: a case-based perspective

**DOI:** 10.1017/ash.2026.10295

**Published:** 2026-02-02

**Authors:** Mustafa Serhat Şahinoğlu, Selcen Özer Kökkızıl, Tuğçe Şimşek Bozok

**Affiliations:** 1 Department of Infectious Diseases and Clinical Microbiology, Manisa City Hospital, Manisa, Türkiye; 2 Department of Infectious Disease and Clinical Microbiology, Mersin University Faculty of Medicine, Mersin, Türkiye

To the Editor,

We read with interest the study by Vathy et al. evaluating the impact of an institution-specific treatment algorithm on the management and outcomes of infections caused by carbapenem-resistant *Acinetobacter baumannii* and *Stenotrophomonas maltophilia* (*S. maltophilia*). The authors report a significant increase in guideline-concordant antibiotic selection and dosing following implementation and education, while secondary clinical outcomes did not significantly change.^1^

This important stewardship work underscores that disseminating evidence-based guidance can meaningfully alter prescribing behavior—yet also highlights the persistent difficulty of translating improved concordance into better patient outcomes for intrinsically resistant organisms.

We would like to contribute a patient-level perspective that may help contextualize this finding for *S. maltophilia*, particularly when disease severity is high and first-line therapy is compromised by resistance.

We recently managed a 71-year-old man with chronic kidney disease on long-term hemodialysis via a tunneled internal jugular catheter, who presented with two weeks of fever and chills occurring during dialysis sessions. Initial evaluation—including transthoracic echocardiography—showed no vegetations. Blood cultures drawn from both catheter and peripheral sites grew trimethoprim–sulfamethoxazole (SXT)–resistant *S. maltophilia*. After early source control with catheter removal and initiation of levofloxacin, the patient initially stabilized; however, he experienced recurrent bacteremia.

Two weeks into therapy, he developed a new murmur and petechiae; repeat echocardiography revealed new mitral and aortic regurgitation and a 1.3 × 1.2 cm mobile vegetation on the native aortic valve, consistent with infective endocarditis.

Given the confirmation of native valve endocarditis, therapeutic options were reassessed. In our clinical setting, access to several alternative agents recommended in international guidance was limited, which influenced antimicrobial selection. Minocycline and aztreonam were not available, and ceftazidime–avibactam was not approved for this indication. Tigecycline was therefore added as a salvage agent within a combination regimen, supported by limited in vitro data suggesting activity and potential synergy against *S. maltophilia*. Despite combination therapy, the patient deteriorated and died from refractory septic shock and multiorgan failure.

This vignette reinforces several stewardship-relevant challenges that may explain why improved guideline adherence does not necessarily yield better outcomes in *S. maltophilia* infection:

1. Evidence gaps in severe disease and endocarditis: Even when clinicians follow existing recommendations, high-quality clinical data to guide regimen choice (monotherapy vs. combination), dosing, and duration in endocarditis remain limited—especially when SXT cannot be used.^2–4^
2. Device-associated infection and diagnostic latency: Our case illustrates how persistent *S. maltophilia* bacteremia in the setting of intravascular devices may indicate an underlying endovascular focus not apparent on initial evaluation, underscoring the need for clinical vigilance and reassessment when bacteremia fails to clear.
3. Potential discordance between in vitro susceptibility and clinical response: Susceptibility-guided therapy may still fail in vivo, particularly in high-inoculum infections, endovascular foci, and patients with major comorbidities.^5^
4. Need for stewardship-enabled decision support: We highlight the potential role of stewardship-supported clinical pathways that clearly define when to pursue echocardiography in persistent *S. maltophilia* bacteremia and how to escalate therapy when first-line agents such as SXT cannot be used.

We commend Vathy et al. for demonstrating that local guidance can improve evidence-based prescribing.^1^ Our case-based perspective suggests that, for *S. maltophilia*, improving outcomes may require not only regimen concordance but also structured diagnostic and escalation strategies, along with stronger clinical evidence for alternative therapies in severe SXT-resistant infections.

We hope this contribution will be useful for stewardship programs refining pathways for difficult-to-treat gram-negative infections.
